# Biological factors and production challenges drive significant UK fruit and vegetable loss

**DOI:** 10.1002/jsfa.13830

**Published:** 2024-09-04

**Authors:** Ewan Gage, Leon A. Terry, Natalia Falagán

**Affiliations:** ^1^ Plant Science Laboratory Cranfield University Bedfordshire UK

**Keywords:** nutrition, challenge, climate change, sustainability, food security

## Abstract

**BACKGROUND:**

Food loss and waste estimates are highly inconsistent as a result of methodological and systemic differences. Additionally, the absence of in‐depth evidence surrounding the biological drivers of food loss and waste precludes targeted mitigation action. To address this challenge, we undertook a metanalysis utilising a systematic literature review combined with industry stakeholder surveys to examine the incidence of food loss and waste in the UK fruit and vegetable supply chain between primary production and retail.

**RESULTS:**

We estimated that 37% of fruit and vegetables, equivalent to 2.4 Mt of produce, is lost between production and sale. In the UK, primary production is the main stage responsible for these losses (58%), and is dominated by four crops (apple, onion, carrot and potato), which contribute 71% of total food loss and waste. Quality and supply/demand mismatch are the core drivers, combined with limited ability to control postharvest quality decline as a result of technical or economic barriers.

**CONCLUSIONS:**

Innate biological mechanisms contribute to, and detract from, marketable quality generating food loss risks where these cannot be adequately modified or controlled. Through climate change effects, reduced pesticide availability, changing consumer behaviour and increased pressure to reduce resource/energy inputs during pre‐ and postharvest handling, food loss and waste risk is likely to increase in the short term unless targeted, coordinated action is taken to actively promote its mitigation. © 2024 The Author(s). *Journal of the Science of Food and Agriculture* published by John Wiley & Sons Ltd on behalf of Society of Chemical Industry.

## INTRODUCTION

Food loss and waste (FLW) accounts for 15% of total carbon dioxide equivalent (CO_2_e) emissions from the food supply chain of the European Union (EU)^1^ alongside posing significant economic and food security risks. Fruit and vegetable products are the largest contributor to global increases in FLW, with 30% of produce lost or wasted across the supply chain in both industrialised and low‐income countries.^2^, ^3^ Fruit and vegetables are highly perishable because they remain biologically active after harvest. Diverse biological origins create heterogenous products with complex postharvest handling requirements, contributing to FLW risk where optimum management is absent because of practical, economic or technical limitations. Postharvest management is commonly applied in a static fashion without accounting for produce variability and dynamic metabolic changes^4^ because pre‐ and postharvest behaviour of fresh produce and its impact on FLW are poorly understood. This leads to a lack of coordinated research bridging physiology with supply chain FLW reduction.^5^ FLW is generally mitigated by infrastructure development, changes to quality standards or policies and improved waste management.^6^, ^7^ Such approaches have limited applicability for fresh produce caused by complex consumer associations between quality and nutritional value,^8^ short shelf‐lives and irregular, unpredictable supply/demand relationships. Knowledge gaps in understanding the biological drivers of FLW in the supply chain currently hinder effective mitigation strategies.

Also, data surrounding FLW risk in fresh produce is inadequate, especially for low volume crops (e.g. raspberry) that have small market shares, but which can contribute to a high cumulative FLW risk. The variations in supply chain length, postharvest infrastructure, quality requirements and handling practice in different markets is generally not captured by the existing literature. Traditional FLW assessment via mass balance or modelling excludes any qualitative evidence relating to root FLW causes.^9^, ^10^ Direct quantification methods such as in‐field assessments may produce high quality evidence. Such methods are resource intensive, often restricting study size to ‘snap‐shot’ surveys, which fail to capture variation within and between seasons, locations, growing systems or cultivars,^11^ particularly for multi‐harvest crops.^12^ This simplification may also limit detailed categorisation of FLW causes, such as generalisation of ‘disease’ rather than in‐depth pathological analysis.^13^ Survey and interview‐based approaches can yield qualitative evidence, although FLW values may be distorted by underestimation and reluctance to disclose information which is difficult to verify.^11^

As a result of these challenges, current FLW evidence is highly polarised between (i) high level quantification lacking detail as to relevant FLW drivers and (ii) highly focused but fragmented analysis that is not representative of the wider supply chain. The present study aimed to use the UK fresh produce supply chain as a case study to identify FLW drivers, based on qualitative evidence. This was achieved through a combination of a systemic literature review with additional evidence gathered by industry stakeholder engagement, allowing demonstration of key FLW causes to guide future mitigation actions.

## MATERIALS AND METHODS

### Literature review

A systematic literature review was performed to identify current estimates of FLW for potato, apple, pear, carrot, onion, cabbage, broccoli/cauliflower, tomato, sweet pepper, cucumber, lettuce, strawberry and raspberry. These crops represent the majority of UK fruit/vegetable market by value/volume^14^ and are sourced from both UK growers and international imports. We exclude import‐only products (e.g. citrus, grape and banana). FLW estimates were included only for studies published since 2010 and that examine conventional intensified production systems utilising agrochemical use and conventional cultivation formats (e.g. field, polytunnel and glasshouse) typical of UK production or major import source based on UK trade statistics, chiefly the EU, North Africa and product‐specific import countries (e.g. South Africa and Chile). Only studies from regions with comparable production and supply chains to the UK were included (EU, North America, New Zealand/Australia, China, Japan and Republic of Korea). Where FLW estimates were provided in sufficient detail, these were allocated to core supply chain phases of (i) harvest losses, (ii) storage, (iii) handling (including packing and local transport) and (iv) retail (comprised of mainstream supermarket sales).

An initial search was carried out using keywords of ‘*x food loss* OR *waste*’ in Google Scholar and Scopus, where *‘x’* was substituted for each product examined followed by generic keywords of ‘*fruit*’, ‘*vegetable*’, ‘*supply chain*’, ‘*primary production*’ and ‘*farm*’, together with reference tracking to identify records. A review of records in the United Nations Food and Agriculture Organisation FLW Database was also employed using country and crop as initial search factors.^15^ After initial screening for studies, which included FLW quantification, 126 FLW sources were identified from both the literature review and the FAO database. Screening of abstracts against the inclusion criteria (country, production system type, methodology, assignment of loss to suitable system boundaries, alignment with FLW definition, duplication) generated 47 studies, of which 37 were retained for analysis following secondary review of the methodology and content (see Supporting information, Table S1). Losses attributed to quality control, processing and transport were included in the (iii) handling category. After evaluation, 401 individual FLW estimates were obtained.

### Stakeholder survey

Knowledge gaps identified by the systematic literature review were used to develop a survey based on semi‐structured interviews with food industry stakeholders (see Supporting information, Table S2). A total of 18 interviews were conducted between April and September 2022 with respondents at managerial/director level in the supply chain. The survey was designed to provide current estimates for FLW in the UK supply chain, identify key FLW drivers and assess sentiment regarding emerging influences on FLW. Our definition of FLW is derived from the Food and Agriculture Organization's definition as a decrease in food volume or quality in produce intended for human consumption within our system boundary between harvest and sale, including inedible parts where integral to marketed products (e.g. stalks/peels). Produce not used for its original purpose was included because FLW, even when it is retained within the food chain as supplementary production is still required to replace FLW, incurring economic/environmental costs.

**Figure 1:**
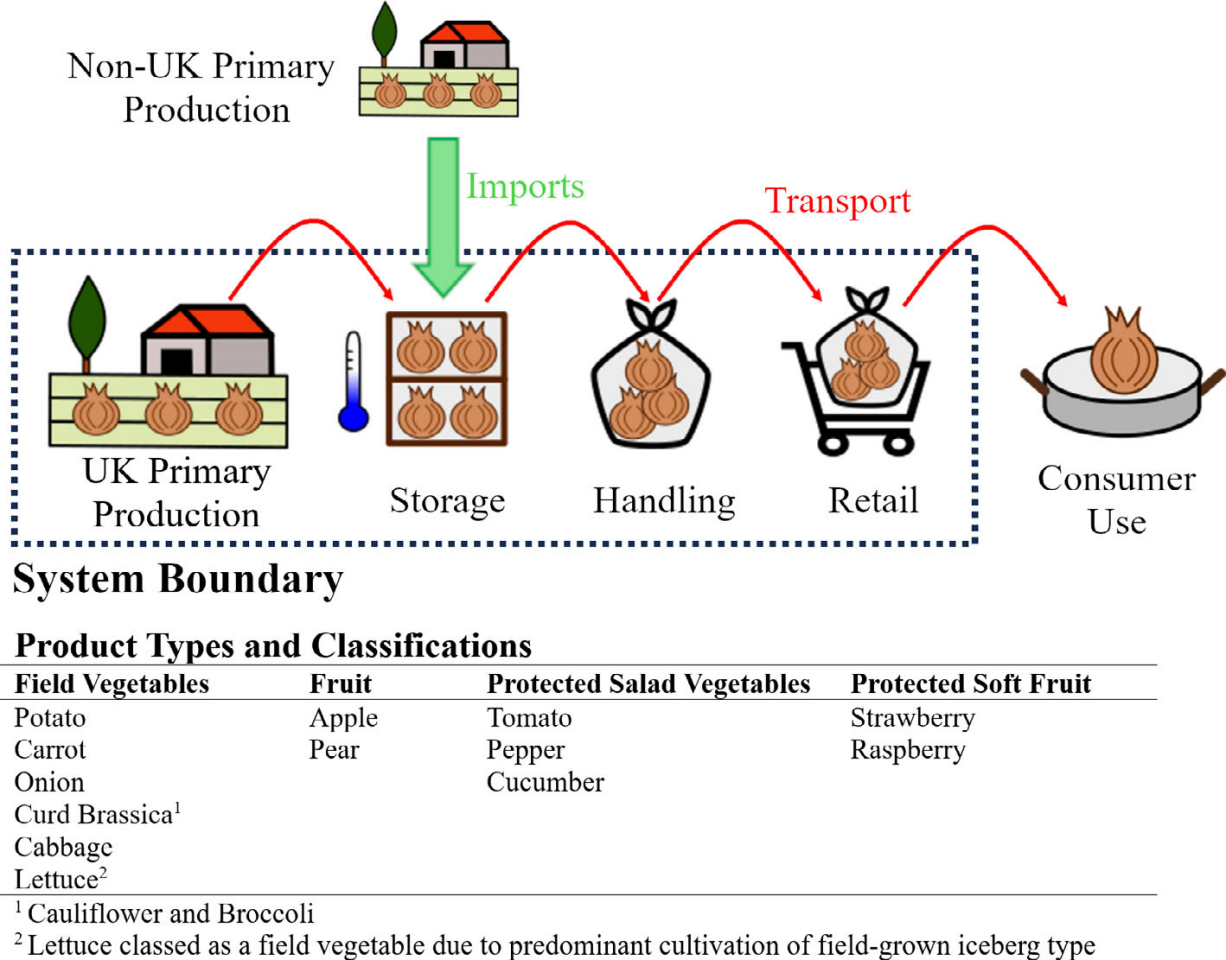
System boundaries used for food loss and waste quantification, including product types covered and their classifications. Produce grown in the UK was examined from harvest (primary production) to retail, whereas imported produce was only included from storage in the UK supply chain.

The system boundaries cover harvest at primary production through to retail, although imported produce was considered only from the point of storage in the UK supply chain (Fig. 1). Pre‐harvest factors were included where relevant to FLW risk higher in the supply chain such as factors increasing postharvest disease development. Interviews were recorded and transcribed into a report, which was confirmed by each respondent prior to further analysis. Interviews were encoded using NVivo 12 (QSR International Pty Ltd, Burlington, MA, USA) for thematic analysis.^16^ Data trends were coded using themes identified in the literature review and developed during the coding process itself. The coding framework was reviewed to define core themes in the survey responses, followed by consolidation of codes to aid identification of FLW causes. Causal mapping was performed to identify interconnected FLW risks.^17^

### Extrapolating UK horticultural FLW

Combined results from the survey and literature review were used to calculate average FLW values for each crop/supply chain stage, which were then used to estimate annual FLW based on home production and import statistics.^14^ Home production values were assumed to be net of harvest FLW. Harvest FLW estimates were only calculated from UK production because representative FLW estimates were obtained only for production systems typical of the UK market, and it was not possible to allocate production system types (and therefore FLW incidence) to imported produce. UK production is orientated towards fresh consumption rather than processing because of low processing value, and so all produce was treated as marketed through retail for FLW estimation, except for potato, where UK retail sales were 25% of total UK supply.^18^

## RESULTS AND DISCUSSION

### Estimating FLW in the horticulture supply chain: the challenge

**Figure 2:**
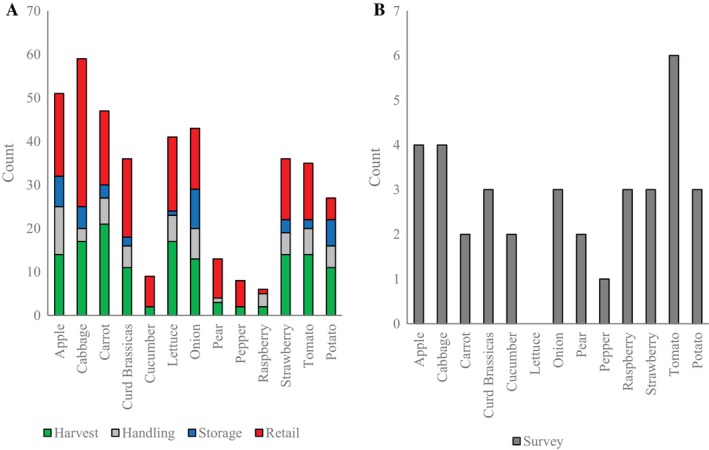
Total counts of food loss and waste estimates for each crop and supply chain stage identified in the literature review (A) and the respondents interviewed for stakeholder survey (B). The survey covered harvest, handling and storage stages only.

The number of published estimates varied significantly between stage and crop type (Fig. 2A). Harvest and retail stages showed the greatest number of reported values and, although most crops were well represented at these stages, low volume crops were only covered in a limited number of studies with raspberry, pepper and cucumber only quantified at harvest by two studies each. The survey covered all crop types besides lettuce, and all supply chain stages besides retail. Respondents generally covered harvest storage and handling activities (Fig. 2B).

**Figure 3:**
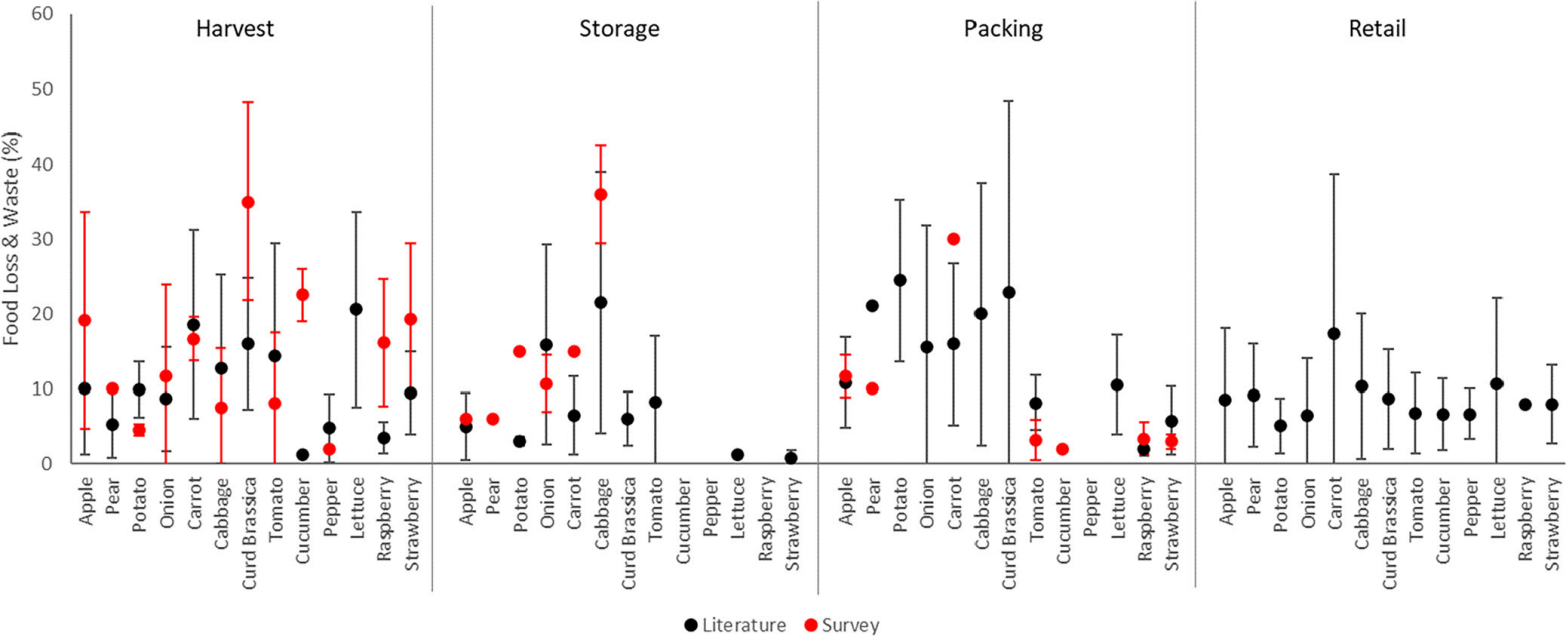
Estimates of food loss and waste in percentage (%) for horticultural crops at harvest, storage, packing and retail as indicated by the literature review and stakeholder survey. Bars indicated the SD of estimates.

Published results showed a limited number of estimates, commonly a source of uncertainty,^19^ and strong bias towards common high‐volume crop types. FLW estimates were highly variable, both from published figures and from the survey for all crops and supply chain stages (Fig. 3). Highly heterogenous food systems make horticultural produce vulnerable to inaccuracy created by small samples sizes, even when derived from a unified estimation methodology,^11^, ^20^ alongside innate variability in FLW between different system types, such as between field and protected cultivation of the same crop type.

Survey estimates generally correlated with published figures, although estimates for apple, carrot and cabbage significantly exceeded published values. Greater FLW reported in UK‐grown crops compared to the literature may be caused by a lack of alternative markets for these products in periods where consumer demand is reduced.

### Product type and supply chain stage: contributions

**Figure 4:**
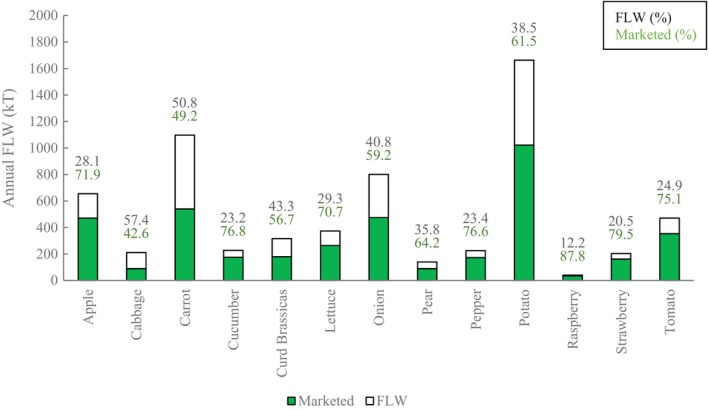
Annual food loss and waste (FLW) and total marketed supply in kilotonnes per year (kT/yr). Data labels correspond to FLW (black) and marketed produce (green) as percentage of total supply.

**Figure 5:**
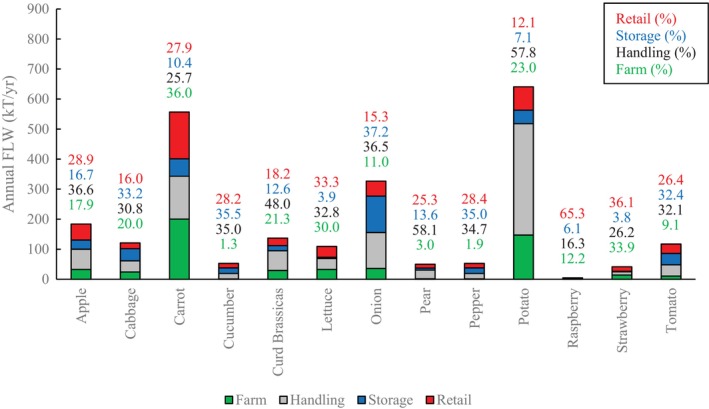
Total annual food loss and waste (FLW) in kilotonnes per year (kT/yr) for fresh produce in the UK supply chain, broken down by stage. Data labels correspond to the percentage loss at each stage.

Total annual FLW of horticultural products in the UK was estimated to be 2395 kilotonnes per year, or 37% of total supply (Fig. 4). A total of 71% was contributed by four crops: apple, onion, carrot and potato. When examined by supply chain stage, the greatest proportion of FLW occurred during primary production (Fig. 5), which contributed 1486 kT of FLW and 58% of total FLW generation. This was predominately a result of the imposition of quality standards during harvest, supply/demand mismatch or the influence of pre‐harvest risks, corresponding with other cross‐supply chain studies.^21^ Increasing ‘premiumisation’ of fresh produce has been reported for the majority of products (even traditionally low‐value products such as potato), driving the effect of quality standards during primary production.^22^

### The origins of FLW in fresh produce by stage

Findings of the survey were placed in juxtaposition with evidence obtained from the literature review to facilitate FLW cause identification. Detailed analysis of survey findings for each crop are presented in the Supporting information (Table S3) and are highlighted below to differentiate from findings from the literature review.

#### FLW during primary production: the quality quandary

Quality is a leading driver of FLW, with produce rejected because of a perceived lack of marketable quality at harvest or after quality declines in the supply chain when assessed against customer specifications. Quality characteristics include size, colour, flavour, texture and abundance of bioactive compounds (e.g. antioxidants) together with an absence of pest/disease (P&D) or mechanical damage. Pre‐harvest conditions and dynamic postharvest processes (e.g. ripening and senescence) modify quality attributes, creating a biological foundation to FLW risk where unmarketable produce results from suboptimal conditions before or after harvest.

##### Quality and supply/demand mismatch as a key driver

Meaningful separation of harvest and handling FLW is precluded for many crops because of differences in location/timing of quality specification imposition. Quality grade out may occur during field harvest for selectively harvest crops (e.g. apple) or during packing for non‐selective mechanically harvested crops such as onion (although some harvesters can be set on minimum bulb size) (see Supporting information, Table S3). Therefore, harvest and handling were treated as a combined process defined as primary production, which varied from < 10% (protected salad vegetables) to 30–40% (field vegetables) and was driven by produce quality and supply/demand mismatch.^21^ Leading subdrivers of quality were weather, P&D and labour access in common with past reports.^23^, ^24^ Specifically, insufficient labour access increased the risk of unharvested produce because of walk‐by losses or lack of economic viability and hindered effective crop management required for optimum quality, particularly in labour‐intensive crops where adequate automation is currently unavailable (e.g. soft fruit). Quality is both a product of innate properties of produce and customer‐derived criteria, although both aspects can be influenced by supply and demand relationships. Quality criteria were reported to be relaxed under periods of undersupply, whereas oversupply was liable to increase specification strictness.

##### Quality specifications increase risk of FLW

Survey estimates for FLW in soft fruit (16.2% strawberry and 19.3% raspberry) exceeded previously published UK figs (2–4%^21^), although they corresponded with other EU studies.^7^ Increasing UK specification strictness from retailer differentiation was described; for example, an increase in minimum premium strawberry size of 2 mm outgrades by 2–3% and risked an additional 10% FLW if this were applied to standard lines. High FLW also results from the use of vulnerable cultivars, which are unsuited to UK conditions to match customer demand (see Supporting information, Table S3). Breeding can help mitigate this issue. Historically, up to 70% FLW in some apple varieties resulted from poor colour development, although this has been reduced to 10% in younger clonal lines. Survey results indicated that historic trends in increasing specification strictness were likely to continue as a result of retailers seeking differentiation in a saturated market environment or by growers in competition with cheaper imports. However, lowering quality standards is unlikely to achieve meaningful FLW reduction. Survey feedback described increased customer complaints where specifications were reduced following shortages, demonstrating high expectations are seen in UK consumers. Consumer misconceptions that attribute lower value to imperfect produce^25^ will hinder customer acceptance of lower quality produce. This generates expectations of matched quality/prices reductions,^26^ and survey feedback was that ‘wonky veg’ schemes had been co‐opted into low‐price offerings rather than targeted solutions for FLW. Reductions in standard product lines with increasing prominence of ‘value’ products was seen in fruit and vegetables following the 2008 recession,^22^ but this has not translated into a corresponding reduction in quality expectations. Longer‐term interventions to modify consumer expectations through education may be a key action for FLW reduction,^26^ but prevention of FLW from within the supply chain is likely to have the greatest, and most immediate, impact.

##### Inherent variability means inherent FLW

Weather variability, as well as unpredictable P&D activity, create suboptimal growing conditions for fresh produce. Climate variation can disrupt field grown produce development preventing marketable quality from being reached, such as calcium nutrition imbalance contributing to blossom end rot in tomato^27^ (see Supporting information, Table S3). Field vegetables experience the greatest variability, such as varied floral initiation linked to water, nitrogen and temperature changes impacting broccoli head quality through size and shape distortions,^28^ contributing to the 35% FLW reported in the survey. Increasing unpredictability of seasonal weather patterns with climate change is likely to intensify FLW risk (see Supporting information, Table S3).

Growing locations and different seasons affect physiology, impacting on quality, storage potential and shelf‐life. For example, fruit size varied by 9.2% with location and 11.1% between seasons in a single apple orchard, which correlated with flower number (and potentially sink/source balance during ripening^29^). Temporal effects can also be significant for tomato, where losses were reported in the survey to vary from 4–30% as a result of environmental changes such as low light in the early season.^11^

Minimising environmental variation may reduce FLW, such as protected cultivation,^7^ but this will be limited by technical and economic barriers. Strawberry loss was reduced from 30% to 8–12% by using polytunnels according to survey results. Protected cultivation will not be feasible for low value, single cut crops (e.g. brassicas, carrots^30^). Even with increased control, significant FLW may still occur, such as the 22.5% FLW reported for cucumber. Cucumber has increased biological risk because it is subject to multiple plantings in a single year, increasing age‐related variation as a result of changes in fruit load and disease pressure^31^ (see Supporting information, Table S3). Protected cultivation will not mitigate the effects of extreme weather events. Heat stress will reduce pollen viability, reducing fruit quality.^32^ Cultural control such as fruit pruning can promote optimum quality through light and microclimate management,^33^ although these may only be impactful in tree, or vine crops and will incur high labour costs. Field vegetables are exposed to the greatest environmental variation, have lower value, and limited control on environmental conditions. Cost‐effective actions are needed to achieve meaningful FLW reductions here. Biological solutions, such as crop breeding, may have minimal economic impact at the same time as reducing FLW through increasing resistance to abiotic stress (e.g. drought), P&D activity or crop uniformity.

#### Storage: creating suboptimal conditions

Storage losses were relatively minor (> 10%) for most crops in the UK. FLW in this part of the supply chain is typically because of a failure to delay senescence, particularly overripening or dormancy break in ware potato and onion. The major storage losses reported were seen only in longer‐term storage crops such cabbage (36%) and onion and potato (both 15%). Cabbage was field or cold stored with trimming waste losses resulting from leaf senescence or disease proliferation (*Botrytis cinerea*) (see Supporting information, Table S3). Storage requires cold conditions, which are energy intensive and force the supply chain to choose between facing economic losses through food spoilage or higher energy prices. Advanced postharvest techniques (e.g. ethylene control) can support the cold chain, allowing the increase in temperature. However, this requires specialised knowledge in postharvest behaviour of fresh produce.

#### Retail: under pressure

Besides oversupply, process control failure, and mechanical damage from handling,^34^ FLW during supermarket retail may result from suboptimal conditions during storage and display, which promote quality loss;^35^ stimulating senescent discoloration, overripening and softening from water loss. However, low‐value/high‐volume crops such as carrot and lettuce may have lower prioritisation for optimal storage and display. This could be a result of a shortage of shelf space,^36^ or conflicting marketing requirements. Survey respondents described marketing changes to increase product visibility leading to loss of quality, such as exposed shelving display increasing potato greening risk. Technological solutions such as modified atmosphere packaging are not extensively used because if cost barriers, and poor control in the retail environment reduces their effectiveness.^37^ Mixed‐product spaces further risk suboptimal conditions through exposure to stressors such as over/under‐chilled storage,^38^ or elevated ethylene levels.^39^

Accurate quantification of retail FLW has been difficult because of a lack of available data and evidence on variable retail conditions, as well as horticultural product responses. Mechanisms that improve resistance to stress in the retail environment (e.g. oxidative stress from high light or low temperature conditions) could further reduce FLW by retarding senescence.

### Navigating the impact of physiology on produce quality

Suboptimal pre‐harvest conditions lead to out of size/shape produce, especially when stress periods overlap with key developmental periods^40^ as a result of distributions in biomass accumulation and partitioning. Water availability was described as a top risk for onion and brassica crops, with increasingly severe summer drought periods reducing quality where insufficient irrigation facilities are available (see Supporting information, Table S3). Unfavourable light and temperature conditions can trigger malformed produce through disruptions of cell division during fruit initiation or uneven pollination in tomato and cucumber.^41^, ^42^ In other crops (e.g. apple), canopy and plant density management can be used to module microclimates, but high labour costs can preclude their use. Environmental control measures, such as manipulating light through truss pruning, can also be used for protected crops to control fruit size.^43^ Because of labour costs/availability, these will only be utilised where distribution modifications are required to avoid large‐scale wastage. The cost/benefit trade‐off means that size/shape driven FLW will always be present in the supply chain, particularly where alternative processing options are unviable.

Biochemical changes during ripening are responsible for the development of fruit colour through metabolism of pigments such as chlorophylls, carotenoids and anthocyanins.^44^ The evolution of taste and flavour profiles through changes in organic acid, sugar and volatile concentrations^45^ will also occur. Physical changes will contribute to fruit softening via weakening of cell wall strength and integrity through cellulose and pectin catabolism.^46^ Quality specifications will dictate produce is supplied in a narrow window of ripeness, with under‐ or overripe produce rejected based on colour or texture (see Supporting information, Table S3). Structural changes, such as the development of fruit abscission zones,^47^ may also contribute to FLW risk where fruit retention is required such as in on‐the‐vine tomato. Failure to control biological mechanisms underpinning maturity will lead to extensive FLW.

Overripening, particularly colour break and excessive softening, is risked where excessive residency times results from delayed sale because of the oversupply or holding of produce to achieve optimum price. In tomato, losses of 5–7% after 4 days of storage, rising to 20% of after days, were reported in periods of oversupply (see Supporting information, Table S3). Harvests can be delayed prolonging fruit lifespan, but this may compromise future harvests because of altered sink/source relationships. Uneven ripening will also result in increased FLW risk, particularly in on‐the‐vine tomato, which generally entails overripe fruit at the truss base or underripe fruit at the top of the truss (see Supporting information, Table S3). Overripening risk may also be increased in response to greater reliance on imported produce because of lengthening supply chains.

Similar to ripening, significant quality changes during dormancy break of perennating organs (onion, potato) generate FLW through changes in taste, texture and appearance, and increased elevated disease risk.^48^ This was reported to contribute up to 10% loss for potato during storage. Sprouting risk will be influenced by internal regulatory mechanisms such as abscisic acid homeostasis and non‐structural carbohydrates,^49^, ^50^ and by pre‐harvest conditions, together with varietal susceptibility, which interact to determine the ability of stored produce to maintain dormancy.^51^, ^52^ Dormancy can be promoted by appropriate handling such as chilling and modulation by exogenous hormonal applications of ethylene and/or 1‐methylcyclopropene.^53^ Chemical treatments such as maleic hydrazide and chlorpropham can be used to prolong dormancy, but use may be restricted by customer requirements or regulatory changes.

However, increased weather variation as a result of climate change, together with demands to reduce costs from energy‐intensive postharvest handling (e.g. onion curing and refrigeration) may contribute to increased FLW, because of a reduced ability to control dormancy. Recent deregistration of chlorpropham in the UK was reported to have increased potato spouting losses by 5–10% and, although alternative treatments are available, these were not considered economically viable for most product lines (see Supporting information, Table S3).

The ability of current postharvest techniques such as controlled atmosphere to delay ripening, dormancy and senescence does not correspond with the need to store produce, particularly in periods of supply/demand mismatch, where FLW of up to 25% may occur for highly perishable crops such as strawberry. Where solutions are available, these may not be economically viable for high volume/low value crops such as onion, particularly given ongoing pressures to reduce energy usage. This FLW risk is inevitable because of UK supply chain dynamics, and improved product postharvest life is necessary to mitigate it. Greater ability to predict produce behaviour in storage to plan marketing and improve postharvest control where possible is necessary to offset this risk. Further postharvest development and biological solutions such as breeding will be essential,^54^ although survey sentiment indicates breeding is reactive to industry needs only in some crops, and focuses on conventional agronomic priorities, not postharvest longevity.

### Pest and disease activity

P&D impact is created by a limited number of agents^11^, ^24^ (Table 1). Field vegetables are particularly vulnerable, with up to 25% harvest loss of broccoli, 15% of carrot and 30–35% in cabbage. Postharvest FLW linked to P&D is also significant, especially for onion rot development (8–9%) (see Supporting information, Table S3). Climate change can contribute to changes in P&D biology such as increase swede midge activity.^55^ Tissue degradation was a leading cause of P&D‐related rejection, alongside cosmetic damage, such as potato skin lesions caused by *Colletotrichum coccodes* or nematode forking of carrot **(**see Supporting information, Table S3).

**Table 1:** Pest and diseases identified as significant contributors to FLW from the stakeholder survey

Crop	Pest	Disease
Brassica	*Contarinia nasturtti*	*Botrytis*
		*Phytopthora infestans*
Carrot	*Meloidogyne* ssp.	*Pythium violae*
Onion		*Fusarium*
		Bacterial rots
		*Botrytis allii*
Potato	*Agrioties sputator*	*Heminthosporium solani*
		*Colletotrichum coccodes*
		*Streptomyces scabies*
	*Globodera* ssp.	
Cucumber	*Frankliniella occidentalis*	*Mycospharella melonis*
Tomato	*Liriomyza*	Tomato Brown Rugous Fruit Virus
	*Tuta absoluta*	*Botrytis*
	*Trialeurodes vaporariorum*	
Raspberry/strawberry	*Drosophila suzukii*	*Podosphaera aphanis*
	*Frankliniella occidentalis*	
	*Tetranychus urticae*	
		*Botrytis*
Apple and pear	*Cydia pomonella*	*Neonectria distissima*
	*Psylla pyricola*	*Pytophthora*
		*Venturia inaequalis*

Storage disease losses were also associated with prolonged residency times, with apple rots increasing to 3–6% of apple stored long‐term to May. Some P&D FLW may be generated by supply chain processes, such as high FLW to *Pythium violae* activity in field‐stored carrot. Beside specific pathologies/agronomic risks, commonalities of FLW risk from P&D include lack of effective control options, favourable weather conditions (warm, humid weather), inoculum availability (poor crop hygiene, field infestation and alternative hosts) or crop vulnerability such as from suboptimal nutrient management.^56^

Further pesticide reductions for environmental or human health reasons will risk yield reductions of 20–40%.^57^ Survey sentiment was that FLW will increase if the ability to actively control P&D continues to decline without corresponding reductions in quality specifications by customers. This risk may vary between crop types. Soft fruit and tomato growers reported more optimism for reduced pesticide availability, whereas field vegetable growers expressed greater concern because of the lack of suitable alternative control options.

Environmental drivers for pesticide reduction need to be reconciled with demands for food security, price and quality.^58^ Alternative methods (e.g. gene editing) may reduce pesticide requirements, although consumer acceptance may hinder implementation, increasing FLW risks.^59^ Breeding timelines of 10–15 years were reported for some products, limiting the ability for breeding to respond to sector needs, highlighting the need for breeding programmes to be accelerated.^60^ Choosing environmental benefits over cutting pesticides may increase FLW unless equally or more effective P&D control methods are adopted.

## CONCLUSIONS

The key causes of FLW were related to crop management during production, lack of optimal postharvest strategies and stock management, and economic, practical and biological challenges. Efforts to reduce FLW in the food system involve understanding its physiological origins for targeted intervention. Agronomic and breeding approaches, systemic improvements and consumer education play crucial roles in the UK context. Although postharvest biology is well documented, its relationship with FLW risk in the supply chain needs further research for effective mitigation. Inevitable FLW arises from inherent growing conditions and P&Ds, potentially increased by current limitations on pesticide use. Despite minor contributions to total FLW in the UK postharvest supply chain, changing demands need new, energy efficient postharvest management approaches that meet fresh produce biological requirements. Addressing supply/demand mismatches and adopting dynamic, biologically‐informed solutions are essential. However, a lack of cohesive evidence on FLW incidence and drivers remains a key barrier. A clear understanding of the biological origins of quality, storability, and FLW risks is crucial for developing impactful solutions and achieving meaningful change in the fresh produce supply chain.

## Supporting information


**Table S1.** Studies utilised in food loss and waste literature review.

**Table S2.** Stakeholder survey used for data collection from supply chain stakeholders.

**Table S3.** Summary of food loss and waste (FLW) causes identified by the stakeholder survey. FLW categories identified by the survey have been graded from minor (+) to major (+++) contributors of FLW for each crop, with each instance assigned to harvest (H), packing/handling (P), storage (S) or retail (R).

## Data Availability

The data that supports the findings of this study are available in the supplementary material of this article.
