# Supplementary material for: Biological factors and production challenges drive significant UK fruit and vegetable loss
Source: J Sci Food Agric. 2024 Sep 4;105(4):2109–17. doi: 10.1002/jsfa.13830 (PMC11824919; doi:10.1002/jsfa.13830)
Supplement: Supplementary file 1 — Table S1. Studies utilised in food loss and waste literature review. [file JSFA-105-2109-s002.docx]

**Supplementary Material 1**

**Table S1.** Studies utilised in food loss and waste literature review.

| 1 | Baker, G. A., Gray, L. C., Harwood, M. J., Osland, T. J., & Tooley, J. B. C. (2019). On-farm food loss in northern and central California: Results of field survey measurements. *Resources, Conservation and Recycling*, *149*, 541-549. |
| --- | --- |
| 2 | Beausang, C., Hall, C., & Toma, L. (2017). Food waste and losses in primary production: Qualitative insights from horticulture. Resources, Conservation and Recycling, 126, 177-185. |
| 3 | Beretta, C., Stoessel, F., Baier, U., & Hellweg, S. (2013). Quantifying food losses and the potential for reduction in Switzerland. Waste management, 33(3), 764-773. |
| 4 | Bond, R. (2016). Carrot Loss during Primary Production (Doctoral dissertation, Master thesis, Hedmark University of Applied Sciences), Retrieved from https://core. ac. uk/download/pdf/52134916. pdf). |
| 5 | Buzby, J. C., Bentley, J. T., Padera, B., Ammon, C., & Campuzano, J. (2015). Estimated fresh produce shrink and food loss in US supermarkets. *Agriculture*, *5*(3), 626-648. |
| 6 | Buzby, J. C., Hyman, J., Stewart, H., & Wells, H. F. (2011). The value of retail‐and consumer‐level fruit and vegetable losses in the United States. *Journal of Consumer Affairs*, *45*(3), 492-515. |
| 7 | Campbell, D., & Munden-Dixon, K. (2018). On-farm food loss: Farmer perspectives on food waste. The Journal of Extension, 56(3), 23. |
| 8 | Eriksson, M., Strid, I., & Hansson, P. A. (2012). Food losses in six Swedish retail stores: Wastage of fruit and vegetables in relation to quantities delivered. *Resources, Conservation and Recycling*, *68*, 14-20. |
| 9 | Esenyan, A. (2014). Want All, Waste All: Exploring Food Waste in the United States. |
| 10 | Franke, U., Hartikainen, H., Mogensen, L., & Svanes, E. (2016). *Food losses and waste in primary production: Data collection in the Nordic countries*. Nordic Council of Ministers. |
| 11 | Ghinea, C. (2017). Assessment of environmental impact of food waste: A case study apple fruits. *Food and Environment Safety Journal*, *16*(1). |
| 12 | Goossens, Y., Berrens, P., Custers, K., Van Hemelryck, S., Kellens, K., & Geeraerd, A. (2019). How origin, packaging and seasonality determine the environmental impact of apples, magnified by food waste and losses. The International Journal of Life Cycle Assessment, 24, 667-687. |
| 13 | Hartikainen, H., Kuisma, M., Pinolehto, M., Räikkönen, R., & Kahiluoto, H. (2014). Food waste in primary production and food processing. *Abstract on results from the Finnish'Foodspill*, *2*. |
| 14 | Hartikainen, H., Mogensen, L., Svanes, E., & Franke, U. (2018). Food waste quantification in primary production–the Nordic countries as a case study. *Waste Management*, *71*, 502-511. |
| 15 | INCOME Consulting - AK2C - 2016 - Food losses and waste: the inventory and their management by stages of the food chain - Report – 164 pages. Report available online (in French) at: https://librairie.ademe.fr/dechets-economie-circulaire/2435-etat-des-lieux-des-masses-de-gaspillages-alimentaires-et-de-sa-gestion-aux-differentes-etapes-de-la-chaine-alimentaire.html. Accessed 13/7/22 |
| 16 | Joensuu, K., Hartikainen, H., Karppinen, S., Jaakkonen, A. K., & Kuoppa-Aho, M. (2021). Developing the collection of statistical food waste data on the primary production of fruit and vegetables. *Environmental Science and Pollution Research*, *28*, 24618-24627. |
| 17 | Johnson, L. K., Dunning, R. D., Bloom, J. D., Gunter, C. C., Boyette, M. D., & Creamer, N. G. (2018). Estimating on-farm food loss at the field level: A methodology and applied case study on a North Carolina farm. *Resources, Conservation and Recycling*, *137*, 243-250. |
| 18 | Kambo, A. R. B. E. N., & Osmani, M. Y. S. L. Y. M. (2018). An evaluation of apple production waste in the municipalities of Korca and Devoll and some related issues. *Albanian J. Agric. Sci. Special Edition-Procceedings of ICOALS*, 739-45. |
| 19 | Koester, U., Empen, J., & Holm, T. (2013). Food losses and waste in Europe and Central Asia. *Food and Agriculture Organization of the United Nations: Rome, Italy*. |
| 20 | Ludwig-Ohm, S., Dirksmeyer, W., & Klockgether, K. (2019). Approaches to reduce food losses in German fruit and vegetable production. *Sustainability*, *11*(23), 6576. |
| 21 | Mattsson, L., Williams, H., & Berghel, J. (2018). Waste of fresh fruit and vegetables at retailers in Sweden–Measuring and calculation of mass, economic cost and climate impact. *Resources, Conservation and Recycling*, *130*, 118-126. |
| 22 | McKenzie, T. J., Singh-Peterson, L., & Underhill, S. J. (2017). Quantifying postharvest loss and the implication of market-based decisions: A case study of two commercial domestic tomato supply chains in Queensland, Australia. *Horticulturae*, *3*(3), 44. |
| 23 | Mena, C., Terry, L. A., Williams, A., & Ellram, L. (2014). Causes of waste across multi-tier supply networks: Cases in the UK food sector. *International Journal of Production Economics*, *152*, 144-158. |
| 24 | Meyer, C. H., Frieling, D., Hamer, M., & Oertzen, G. (2017). *Food losses in supply chains for fruits, vegetables and potatoes between field and retail shelf in North-Rhine Westphalia, Germany* (No. 1012-2017-670). |
| 25 | O'Connor, T., Kleemann, R., & Attard, J. (2022). Vulnerable vegetables and efficient fishers: A study of primary production food losses and waste in Ireland. *Journal of Environmental Management*, *307*, 114498. |
| 26 | Porat, R., Lichter, A., Terry, L. A., Harker, R., & Buzby, J. (2018). Postharvest losses of fruit and vegetables during retail and in consumers’ homes: Quantifications, causes, and means of prevention. *Postharvest biology and technology*, *139*, 135-149. |
| 27 | Riggi, E., & Avola, G. (2010). Quantification of the waste stream from fresh tomato packinghouses and its fluctuations: Implications for waste management planning. *Resources, conservation and recycling*, *54*(7), 436-441. |
| 28 | Roels, K., Vangeyte, J., Linden, V. V., & Gijseghem, D. V. (2012, July). Food losses in primary production: the case of Flanders. In *Proceedings CIGR-Ag. Eng. 2012: International Conference on Agricultural Engineering, Valencia, Spain*. |
| 29 | Rogers, G., Ekman, J., & Titley, M. (2013). Identifying new products, uses and markets for Australian vegetables: A desktop study. *Horticulture Australia Ltd*, 30-32. |
| 30 | Rohr, S., Mounter, S., Fleming, E. & Griffith, G. (2020). Loss and Waste in the Australian Fresh Apple Value Chain. *Proceedings in Food System Dynamics*, 134-152. |
| 31 | Schneider, F., Part, F., Göbel, C., Langen, N., Gerhards, C., Kraus, G. F., & Ritter, G. (2019). A methodological approach for the on-site quantification of food losses in primary production: Austrian and German case studies using the example of potato harvest. *Waste Management*, *86*, 106-113. |
| 32 | Sheane, R., McCosker, C., & Lillywhite, R. (2017). Food Waste in Primary Production—A Preliminary Study on Strawberries and Lettuces. *Defra SCF0307/WRAP OIN006-001. Available at http://www. wrap. org. uk/sites/files/wrap/Food_waste_in_primary_production_report. pdf*. |
| 33 | Siu, A. (2014). An Analysis of Food Waste in Ontario's Domestic Fresh Strawberry Supply Chain. |
| 34 | Spang, E. S., & Stevens, B. D. (2018). Estimating the blue water footprint of in-field crop losses: A case study of US potato cultivation. *Sustainability*, *10*(8), 2854. |
| 35 | Strid, I., & Eriksson, M. (2014). Losses in the supply chain of Swedish lettuce–wasted amounts and their carbon footprint at primary production, whole sale and retail. In *The 9th International Conference on LCA in the Agri-Food Sector, San Francisco*. |
| 36 | Svanes, E., & Johnsen, F. M. (2019). Environmental life cycle assessment of production, processing, distribution and consumption of apples, sweet cherries and plums from conventional agriculture in Norway. *Journal of Cleaner Production*, *238*, 117773. |
| 37 | Terry, L. A., Mena, C., Williams, A., Jenney, N., & Whitehead, P. (2011). Fruit and vegetable resource maps: Mapping fruit and vegetable waste through the wholesale supply chain. |
| 38 | Thorsen, M., Mirosa, M., & Skeaff, S. (2022). A quantitative and qualitative study of food loss in glasshouse-grown tomatoes. *Horticulturae*, *8*(1), 39. |
| 39 | Vinyes, E., Asin, L., Alegre, S., Muñoz, P., Boschmonart, J., & Gasol, C. M. (2017). Life Cycle Assessment of apple and peach production, distribution and consumption in Mediterranean fruit sector. *Journal of Cleaner Production*, *149*, 313-320. |
| 40 | Willersinn, C., Mack, G., Mouron, P., Keiser, A., & Siegrist, M. (2015). Quantity and quality of food losses along the Swiss potato supply chain: Stepwise investigation and the influence of quality standards on losses. *Waste management*, *46*, 120-132. |
| 41 | Winans, K., Marvinney, E., Gillman, A., & Spang, E. (2020). An evaluation of on-farm food loss accounting in life-cycle assessment (LCA) of four California specialty crops. *Frontiers in Sustainable Food Systems*, *4*, 10. |
| 42 | Xiangyang, W., & Bagshaw, J. S. (2001). Postharvest handling systems assessment of pak choi and Chinese cabbage in Eastern-central China. *Postharvest Handling of Fresh Vegetables*. |
| 43 | Zheng, S., Wu, L., Gao, L., & Wu, P. (2000). Assessment of postharvest handling systems of vegetable crops in the Beijing Area. *Preface 11 Opening Address 12*. |
| 44 | Borum, A., Mogsensen, L., Kristensen, T., Hammershøj, J. R., Bertelsen, M. G. (2018) Food Waste in the Danish primary production and food industries. Available online at: https://dcapub.au.dk/djfpublikation/djfpdf/DCArapport143_1.pdf Accessed 21/7/22 |
| 45 | Hayhoe, M. A. (2019). *Exploration into Food Waste Occurring in Bovine Dairy, Leafy Green and Apple Supply Chains* (Doctoral dissertation, University of Guelph). |
| 46 | Plazzotta, S., Manzocco, L., & Nicoli, M. C. (2017). Fruit and vegetable waste management and the challenge of fresh-cut salad. *Trends in food science & technology*, *63*, 51-59. |
| 47 | Snow, T., and E. Dean. "Food loss in Vermont: Estimating annual vegetable & berry loss. A salvation farms’ analysis." (2016). |
