# Supplementary material for: Biological factors and production challenges drive significant UK fruit and vegetable loss
Source: J Sci Food Agric. 2024 Sep 4;105(4):2109–17. doi: 10.1002/jsfa.13830 (PMC11824919; doi:10.1002/jsfa.13830)
Supplement: Supplementary file 2 — Table S2. Stakeholder survey used for data collection from supply chain stakeholders. [file JSFA-105-2109-s001.docx]

**Supplementary Information 2**

Stakeholder survey used for data collection from supply chain stakeholders.

**ZECC Stakeholder Survey for Food Loss & Waste Hotspot Identification**

**Survey –** Version 1.5, 29/3/22

| **Name** | |
| --- | --- |
|  | |
| **Company & Position** | |
|  | |
| **Address** | |
|  | |
| **Contact Details (Email, Telephone Number)** | |
|  | |
| **Survey Format** | |
| Online  Telephone  Face-to-Face  Other  **Survey Recorded?** | |
| **Interview Date & Time** | |
|  | |
| **Interview led by:** | |
|  | |
| **Presurvey Activities** | |
| Has the interviewee been presented with the participant information sheet, and completed the survey consent form (and NDA if required)? **Yes  NDA?** | |
| **Survey Number** |  |

1. **About Your Business**

| **1.1 What are your main activities in the supply chain?** |
| --- |
|  |
| **1.2 Who are your major customers?** |
|  |
| **1.3 Do you supply only this customer?** |
|  |

1. **About Your Fresh Produce**

| **2.1 Principal product types** |
| --- |
|  |
| **2.2 What are your annual production/sales (volume and value) for these products?** |
|  |
| **2.3 What proportion of this is imported? And from where?** |
|  |
| **2.4 What is your market share for these products?** |
|  |
| **2.5 How variable is demand? When are your windows of supply/demand?** |
|  |
| **2.6 Are you able to smooth out supply and demand variation? If so, how?** |
|  |
| **2.7 What is your lead time (e.g. hours from order to delivery)?** |
|  |
| **2.8 What is the typical unit size/volume at dispatch?** |
|  |
| **2.9 Can you share any technical details about your products?** |
|  |

1. **Your Ideas about Food Loss & Waste (FLW)**

| **3.1 How do you define food loss and/or waste?** |
| --- |
|  |
| **3.2 Do you record FLW? If so, how and why?** |
|  |
| **3.3 What are your main causes of FLW?** |
|  |
| **3.4 What are your main actions to avoid FLW?** |
|  |
| **3.5 What happens to excess, downgraded or damaged products?** |
|  |
| **3.6 What happens to products that have exceeded their shelf life?** |
|  |
| **3.7 Do you have alternative markets for your produce?** |
|  |

1. **Causes of Food Loss & Waste**

| **Product Details**  NB. Product Limits: If multiple product lines are produced restrict responses below to the greatest volume product line for top 2/3 crop types (or similar if only one type is handled but of multiple cultivars). Replicate and annotate the sections below as required. | | | | |
| --- | --- | --- | --- | --- |
| **Product 1** |  | | | |
| **Product 2** |  | | | |
| **Product 3** |  | | | |
| **4.1 What is your total volume of FLW per year?** | | | | |
|  | | | | |
| **4.2 Which of the following reasons play the most important role in FLW?**  **(Please rank and give rough percentage where possible)** | | | | |
| Grade Out/Specification | |  | Temperature Controls/Records |  |
| Pest/Disease Damage or Presence | |  | Atmosphere Management |  |
| Weather/Climate Damage | |  | Packaging (inc. Traceability Coding) |  |
| Produce Quality at Harvest | |  | Over Supply/Lack of Market |  |
| Handling/Harvest Damage | |  | Customer Behaviour/Rejections |  |
| Postharvest Quality Loss | |  | Food Safety |  |
| Produce Maturity – early/late harvest | |  |  |  |
| **4.3 Where do you see FLW occurring in your business?** | | | | |
| Pre-harvest | |  | Repacking (e.g. retail packing) |  |
| Harvest (e.g. produce left in field) | |  | Storage (dispatch) |  |
| Grading | |  | Storage (store) |  |
| Packing (field stores) | |  | Point of Sale Display |  |
| Storage (field stores) | |  | Other (please Specify) |  |
| Transport | |  |  |  |
| **4.4 Out of these reasons given in 4.1 and 4.2 above, which are the most important/difficult to resolve?** | | | | |
|  | | | | |
| **4.5 Do you see any significant changes in FLW with season/weather changes?** | | | | |
|  | | | | |
| **4.6 What impacts do variety choice and growing conditions have on FLW?** | | | | |
|  | | | | |
| **4.7 How important is country of origin on FLW?** | | | | |
|  | | | | |
| **4.8 Are there any other sources of FLW we have not discussed?** | | | | |
|  | | | | |

1. **Food Loss & Waste Links with Product Specifics**

| **5.1 Are there any specific characteristics of your products that increase FLW risks?** |
| --- |
|  |
| **5.2 What is the impact of product damage on waste for these products?** |
|  |
| **5.3 What is the impact of quality control/product specifications for this product?**  **What are the most common causes of rejection or discard?** |
|  |
| **5.4 At what level are product specifications, variety choice etc. decided?**  **Do you have much ability to impact these criteria?** |
|  |
| **5.5 What parameters do you record to monitor or predict FLW?** |
|  |
| **5.6 Do you feel like compromises have to be made to achieve sufficient shelf life?**  **e.g. reduced ripeness, firmer cultivars at the expense of taste/flavour etc.** |
|  |

1. **Harvest**

| **6.1 What proportion of your crop is not harvested?** |
| --- |
|  |
| **6.2 What are the main causes of this?** |
|  |
| **6.3 Do you act to reduce FLW at harvest?** |
|  |

1. **Storage & Handling**

| **7.1 What nature of produce storage occurs at your business?** |
| --- |
|  |
| **7.2 What is the timeline for product storage?** |
|  |
| **7.3 How much produce do you store at a given time?** |
|  |
| **7.4 Can you estimate the proportion of FLW during storage?** |
|  |
| **7.5 What are your storage conditions?** |
|  |
| **7.6 Do you take any specific action to reduce FLW in store?** |
|  |
| **7.7 Are there any common problems/limits in the storage life of your products?** |
|  |
| **7.8 What is the impact of quality control/product specifications for this product?** |
|  |
| **7.9 Are there any specific stacking/shelving polices for your products?** |
|  |

1. **Processing**

| **8.1 What processing activities (e.g. cutting, cleaning) take place in your business?** |
| --- |
|  |
| **8.2 Can you estimate the proportion of FLW during processing?** |
|  |
| **8.3 What are the causes of FLW during processing?** |
|  |
| **8.4 What is the impact of quality control/product specifications for this product?** |
|  |
| **8.5 Does behaviour elsewhere in the supply chain impact the level of FLW during processing?** |
|  |
| **8.6 Does FLW management have any impact on processing choices?** |
|  |
| **8.7 Do you carry out any certification or H&S testing?** |
|  |

1. **Packing**

| **9.1 What packing activities are performed?** |
| --- |
|  |
| **9.2 Can you estimate the proportion of FLW during packing?** |
|  |
| **9.3 How do you minimise FLW during packing?** |
|  |
| **9.4 What packing types and technologies do you use?** |
|  |
| **9.5 Are there any specific stacking/shelving polices for your products?** |
|  |
| **9.6 Do product specifications contribute to FLW at packing?** |
|  |
| **11.7 Does FLW management have any impact on packing choices?** |
|  |

1. **Transport**

| **11.1 What forms of transport are used for produce moving to/from your business?** |
| --- |
|  |
| **11.2 What are the typical distances between your business and your suppliers?** |
|  |
| **11.3 What are the typical distances between your business and your customers?** |
|  |
| **11.4 What level of loss do you to typically see in the transport stages?** |
|  |
| **11.5 What conditions do you specify during transport?** |
|  |
| **11.6 Are you subject to any maximum transport times?** |
|  |
| **11.7 Do you take any specific action to limit FLW in transit?** |
|  |

1. **Retail**

| **11.1 What is the typical timeline for produce within the supply chain?** |
| --- |
|  |
| **11.2 What level of loss do you to typically see at the retail stage?** |
|  |
| **11.3 How do you forecast your orders?** |
|  |
| **11.4 What conditions do you specify during the retail phase?** |
|  |
| **11.5 Are you subject to any maximum shelf-life periods?** |
|  |
| **11.6 Do you take any specific action to limit FLW at the retail stage?** |
|  |

1. **Marketing Forces**

| **12.1 What is the impact of forecasting on FLW?** |
| --- |
|  |
| **12.2 Does marketing behaviour have any impact on FLW?** |
|  |
| **12.3 What are the impacts of shelf-life and traceability policies on FLW?** |
|  |
| **12.4 Do you routinely forecast to mitigate FLW (e.g. over-order or over-plant?)** |
|  |
| **12.5 What are the penalties for not delivering complete orders on time, or having orders rejected after quality controls?** |
|  |
| **12.6 Could market behaviour be changed to reduce FLW? If so, how?** |
|  |
| **12.6 Have you targeted FLW directly through marketing? Have these been effective?** |
|  |
| **12.8 Has Brexit/Covid-19 or current geopolitics impacted FLW? If so, how?** |
|  |
| **12.9 How does customer/marketing behaviour impact packing-related FLW?** |
|  |

1. **Managing FLW in your Business**

| **13.1 Do you have any active technologies to reduce FLW? If so, what?** |
| --- |
|  |
| **13.2 Which causes of FLW would you prioritise for improvement?** |
|  |
| **13.3 Are there any FLW management techniques you are interested in adopting?** |
|  |
| **13.4 Are there any barriers to adopting new FLW management techniques?** |
|  |
| **13.5 Where would you place FLW relative to other influences on your business?**  **e.g. Labour availability, market conditions, energy etc.** |
|  |
| **13.6 What features would encourage you to reduce FLW?** |
|  |
| **13.7 How much of a driver is sustainability/environmental impact for your business?** |
|  |
| **13.8 Do you link FLW with environmental impacts or the sustainability of your business?** |
|  |
| **13.9 Is FLW included in any environmental or sustainability objectives of your business?** |
|  |
| **13.11 Have you seen any changes in the proportion of FLW over the last 10 years?** |
|  |
| **13.11 Do you foresee any changes in the incidence of FLW in the next 10 years?** |
|  |
