# Supplementary material for: Biological factors and production challenges drive significant UK fruit and vegetable loss
Source: J Sci Food Agric. 2024 Sep 4;105(4):2109–17. doi: 10.1002/jsfa.13830 (PMC11824919; doi:10.1002/jsfa.13830)
Supplement: Supplementary file 3 — Table S3. Summary of food loss and waste (FLW) causes identified by the stakeholder survey. FLW categories identified by the survey have been graded from minor (+) to major (+++) contributors of FLW for each crop, with each instance assigned to harvest (H), packing/handling (P), storage (S) or retail (R). [file JSFA-105-2109-s003.docx]

**Supplementary material 3: Summary of food loss and waste (FLW) causes identified by the stakeholder survey**

FLW categories identified by the survey have been graded from minor (+) to major (+++) contributors of FLW for each crop, with each instance assigned to harvest (H), packing/handling (P), storage (S) or retail (R). hjh

**Field Vegetables**

| **Stage** | **Loss Category** | **Carrot** | **Cabbage** | **Potato** | **Onion** | **Curd Brassica** | **Notes** |
| --- | --- | --- | --- | --- | --- | --- | --- |
| H | Mechanical damage during harvest/handling | + |  | ++ | ++ |  |  |
| H | Quality gradeout during harvest |  | + | + | + | +++ | **Potato/Onion**: Size selection during automated harvesting  **Brassicas**: Selective hand harvests according to quality specifications, although maybe subject to trimming waste in the field. |
| S/P | Quality gradeout after harvest | +++ |  | ++ | ++ |  | Imposition of specification criteria during packing. |
| H/P/S/R | Size/Shape | ++ | + | + | ++ | + | Size/shape FLW risk influenced by availability of alternative processing.  **Cabbage**: May contribute to gross FLW through increased trimming of oversized heads.  **Carrot**: Forking due to nematode action.  **Potato**: Stricter size requirements for premium products (e.g. baking potato)  **Onion**: FLW during harvest due to undersized bulbs can approach 5%. |
| H/S/P/R | Colour |  |  | + |  | + | **Curd Brassicas**: Curd discolouration due to sunlight exposure (cauliflower) or bronzing/yellow beading (broccoli).  **Potato**: Greening during retail periods.  **Onion**: Development of skin colour during curing. |
| H/S/P | Physical condition (e.g. skin texture/thickness, inflorescence condition) |  |  | ++ | ++ | + | **Carrot**: Cavity spot damage, outer skin condition.  **Potato**: Skin blemishes from disease  **Onion**: Thickness, number and entirety of outer skin layers. |
| H/S | Disease | +++ | +++ | +++ | +++ | ++ | **Brassica**: *Botrytis, Phytopthora infestans*  **Carrot***: Pythium violae*  **Potato***: Helminthosporium solani, Colletotrichum coccodes, Streptomyces scabies*  **Onion***: Fusarium oxysporum, Botrytis allii* |
| H/S | Pest activity | +++ | +++ | ++ | +++ | +++ | **Carrot***: Psila rosae, Meloidogyne* ssp.  **Potato***: Agriotes* ssp., *Globodera* ssp.  **Brassicas**: *Contarinia nasturtii* |
| H | Weather/Climate - Damage | + | + |  |  | + |  |
| H/S | Weather/Climate - Interacting Effects (e.g. Disease) |  | +++ | ++ | ++ | +++ | **Cabbage**: Trimming losses increased following disease activity.  **Potato/Onion**: Poor conditions prior to harvest exacerbate storage disease risk, with a 10% increase in rots during wet harvest periods. |
| H/S | Weather/Climate - Maturation/Developmental |  | ++ |  |  | ++ | **Brassicas**: Drought periods impacting achieved size at harvest  **Onion**: Water scarcity during bulb initiation and development can impact size at harvest. |
| S | Physiological Storage Loss - Water loss, senescence |  | ++ | ++ | ++ |  | **Cabbage**: Prolonged storage increases trimming waste due to greater leaf senescence. |
| S | Dormancy Loss |  |  | +++ | +++ |  | **Potato/Onion**: Sprouting/root development following dormancy break, particularly after deregistration of CIPC. |
| H/P | Labour availability/efficacy | + | +++ | + | ++ | +++ | Labour shortages reduce harvest number, and increase risk of walk-by losses. |
|  | Supply/demand mismatch | +++ | +++ | ++ | ++ | +++ | Ability to access alternative markets (e.g. processing) increased with field vegetables, but constraints are present on crops with limited shelf life (e.g. broccoli) which limits actions to match supply with demand, whilst prolonged storage increases loss incidence in other crops (e.g. cabbage). |

**Apples & Pears**

| **Stage** | **Loss Category** | **Apples & Pears** | **Notes** |
| --- | --- | --- | --- |
| H | Mechanical damage during harvest/handling | (+) | Tearing and scarring of fruit base during harvest (apple) or puncturing of skin, compression or dehydration (pear). |
| S/P | Quality gradeout after harvest | ++ | Post-storage gradeout may see 10% losses based on fruit quality based on variety risk and length of storage. |
| H | Quality gradeout during harvest | +++ | Primary imposition of quality standards during harvest, giving around 5% (apple) and 10% (pear) loss at harvest. |
| H/P/S/R | Size/Shape | +++ | Tight specifications for individual fruit size/weight, particularly for vulnerable varieties. |
| H/S/P/R | Colour | +++ | High rejection rates for insufficient colour development, approaching 30% in bad years for vulnerable varieties. |
| H/S/P | Physical condition | ++ | Rejections due to texture/sugar content below minimum thresholds, or poor flavour.  Poor skin condition (russeting). |
| H/S | Disease | ++ | *Nectria galligena* infections can causes losses at harvest through eye rot, whilst rots can develop during prolonged storage that can approach 6%. Apple scab (*Venturia inaequalis*) may result in fruit skin lesions. |
| H/S | Pest activity | + | *Psylla pyricola* honeydew can lead to discolouration of pear fruit. |
| H | Weather/Climate - Damage (e.g. frost) | + | Frost/hail damage can occur during the early season, and contribute to russeting. |
| H/S | Weather/Climate - Interacting Effects (e.g. Disease) | ++ | Frequent wet/dry cycles can increase losses through *Nectria,* whilst hot/wet periods can increase *Venturia* damage. |
| H/S | Weather/Climate - Maturation/Developmental | + | Poor weather can increase postharvest losses through changes in dry matter accumulation. |
| S | Physiological Storage Loss | ++ | Textural changes during storage are key contributors to storage FLW. Watercore (apple core becomes vitrified during storage), textural loss, declines in dry matter. Optimised storage typically prevents off-flavours from fermentation, although internal browning can occur in susceptible varieties. |
| H/P | Labour availability/efficacy | +++ | Insufficient labour access will reduce harvest number, delay harvesting beyond peak maturity or increase walkby loss risk. |
|  | Supply/demand mismatch | +++ | Lack of alternative markets increases FLW risk, especially where large quantities of stored fruit are at risk of becoming unmarketable. |
|  | Variety Choice | + | Some varieties can be difficult to grow to customer specifications, increasing FLW risk e.g. due to insufficient colour development. |

Protected Soft Fruit

| **Stage** | **Loss Category** | **Strawberry &**  **Raspberry** | **Notes** |
| --- | --- | --- | --- |
| H | Mechanical damage during harvest/handling, walkby losses | ++ | c. 10% of marketable fruit can be left in walkby losses, whilst improper handling can result in fruit bruising. Up to 20% of fruit can be lost due to handling/mechanical damage. |
| S/P | Quality gradeout after harvest | (+) | Only limited postharvest losses due to short residency times of around 2-3%. |
| H | Quality gradeout during harvest | +++ | Significant FLW during harvest due to imposition of tight quality specifications (20 – 30%). |
| H/P/S/R | Size/Shape | +++ | Strict size and shape specifications can result in losses of up to 20% at harvest. Fruit size can be impacted by climate, plant age and point in the season. Fruit size/shape can be disrupted by pollinator activity and plant nutritional status. |
| H/S/P/R | Colour | (+) | Only minor losses due to white shoulder (strawberry) or excessive depth of colour. |
| H/S/P | Physical condition | ++ | Fruit texture will reduce later in the season, increasing bruising risk.  Excessive storage will increase softening and may result in juice leakage.  Drupelet adhesion (raspberry) and physical damage may result in rejections. |
| H/S | Disease | +++ | Rots (*Botrytis*) can result in up to 20% loss at harvest, with further losses during storage. Powdery mildew (*Podosphaera aphanis*) may also reduce strawberry quality. |
| H/S | Pest activity | +++ | Fruit damage from *Drosophila suzukii* and *Tetranychus urticae* may render fruit unmarketable. *Frankliniella occidentalis* can result in damage to fruit surfaces (bronzing). |
| H | Weather/Climate - Damage (e.g. frost) |  | Largely avoided through use of protected cultivation. |
| H/S | Weather/Climate - Interacting Effects (e.g. Disease) | +++ | Weather effects on supply/demand curves are difficult to predict due to large changes in consumer demand and yields.  Hot/wet weather will increase rot risk and reduce fruit texture. |
| H/S | Weather/Climate - Maturation/Developmental | ++ | Effect of weather on harvest volume impacts supply/demand mismatch risk. |
| S | Storage Loss | (+) | Storage losses typically only occur as a result of supply/demand mismatch. |
| H/P | Labour availability/efficacy | ++ | Insufficient labour access will reduce crop management abilities, delay harvesting beyond peak maturity or increase walkby loss risk. |
|  | Supply/demand mismatch | +++ | Poor ability to match supply/demand and lack of alternative markets generates significant FLW risk during overproduction periods of up to 25%. Prolonged storage will also increase FLW risk through softening and rots in store. |
|  | Transportation losses | (+) | Minor losses due to logistical disruption/improper temperature management of 1 – 2%. |
|  | Variety Choice | +++ | Customer specifications are for varieties with higher sugar content and softer texture, increasing bruising/rot risk. |

**Protected Salad Vegetables**

| **Stage** | **Loss** |  |  |
| --- | --- | --- | --- |
| H | Mechanical damage during harvest/handling | (+) | **Tomato**: Fruit drop during handling, particularly for baby plum varieties. |
| S/P | Quality gradeout after harvest | (+) | Only minor packhouse loss due to limited storage periods of around 1-2%, although this increases to 20% for longer storage periods. Minor FLW from missed imperfections during harvest. |
| H | Quality gradeout during harvest | ++ | **Tomato**: Minor losses during harvest, with <5% loss at harvest considered optimum, but will be greater for large vine varieties and can be c. 15% on average although some fruit will be harvested green that are unlikely to mature. |
| H/P/S/R | Size/Shape | +++ | **Pepper**: Misshapen fruit rejected at harvest.  **Cucumber**: Significant rejections from curved fruit. |
| H/S/P/R | Colour | ++ | **Tomato**: Overly red tomatoes can be rejected due to overmaturity. Golden Speckle can result from excessive calcium supply.  **Cucumber**: Rejections from overdeveloped dark-green fruit grown under high fruit load.  **Pepper**: Uneven colour development can result in rejections, particularly for yellow-orange-red transitions. |
| H/S/P | Physical condition | + | **Tomato**: Calyx condition (colour, desiccation) and fruit drop from poor retention can lead to loss during harvest or rejection in the supply chain. Fruit splitting. Achievement of required sugar content can be difficult in overwintered production. Sequential truss setting will result in fruit harvested that is either over-ripe or immature.  **Pepper**: Integrity of pointed peppers, or petiole condition of bell peppers. |
| H/S | Disease | (+) | **Tomato:** Generally a minor cause of FLW, but requires careful crop management to minimise losses.  **Cucumber**: *Mycospharella* infections can result in internal fruit rots. |
| H/S | Pest activity | (+) | **Tomato**: Generally a minor cause of FLW, but requires careful crop management to minimise losses.  **Cucumber:** *Frankliniella occidentalis* can give misshapen fruit or skin blemishes.  **Pepper**: Damage to fruit caused by thrips and |
| H | Weather/Climate - Damage (e.g. frost) | + | **Tomato**: Climate conditions can increase fruit splitting risk and vulnerability to blossom end rot. |
| H/S | Weather/Climate - Interacting Effects (e.g. Disease) | + | Weather impacts harvest volumes, particularly in unlit crops. |
| H/S | Weather/Climate - Maturation/Developmental | +++ | **Tomato:** Stress during fruit setting or maturation can result in fruit splitting |
| S | Storage Loss | ++ | **Tomato**: Physical compression damage can occur during long-term storage, together with increased splitting risk. |
| H/P | Labour availability/efficacy | ++ | Reduced labour availability/efficacy limits the ability to manage crop and harvests to optimum requirements. |
|  | Supply/demand mismatch | +++ | Poor ability to match supply/demand through unpredictable harvests and limited ability to hold produce/lack of alternative markets generates significant FLW risk during overproduction periods. Limited ability to hold fruit on plants/in store reduces capacity to match supply with demand. |
|  | Variety Choice | + | Customers can specify varieties prone to FLW due to difficulties achieving specified quality or disease susceptibility. |
